# Supplementary material for: Tailoring the Hydroxyl Density of Glass Surface for Anionic Ring-Opening Polymerization of Polyamide 6 to Manufacture Thermoplastic Composites
Source: Polymers (Basel). 2022 Sep 3;14(17):3663. doi: 10.3390/polym14173663 (PMC9460734; doi:10.3390/polym14173663)
Supplement: Supplementary file 1 [file polymers-14-03663-s001.zip › polymers-1866134-supplementary.pdf]

Supplementary Materials

Tailoring the hydroxyl density of glass surface for anionic ring opening polymerization of Polyamide 6 to manufacture thermoplastic composites

Achraf Belkhiri,[1,2] *Nick Virgilio,*[3] *Valérie Nassiet,*[1] *Hélène Welemane,*[1] *France Chabert,* [1*] *and Olivier De Almeida,*[2]

[1]Laboratoire Génie de Production (LGP), Université de Toulouse, INP-ENIT, Tarbes, France

[2]Institut Clément Ader, Université de Toulouse, CNRS UMR 5312, IMT Mines Albi, UPS, INSA, ISAE-SUPAERO, Campus Jarlard, 81013 Albi, France

[3]CREPEC, Department of Chemical Engineering, Polytechnique Montréal, Montréal, Canada

* France Chabert france.chabert@enit.fr

1. Reaction mechanisms

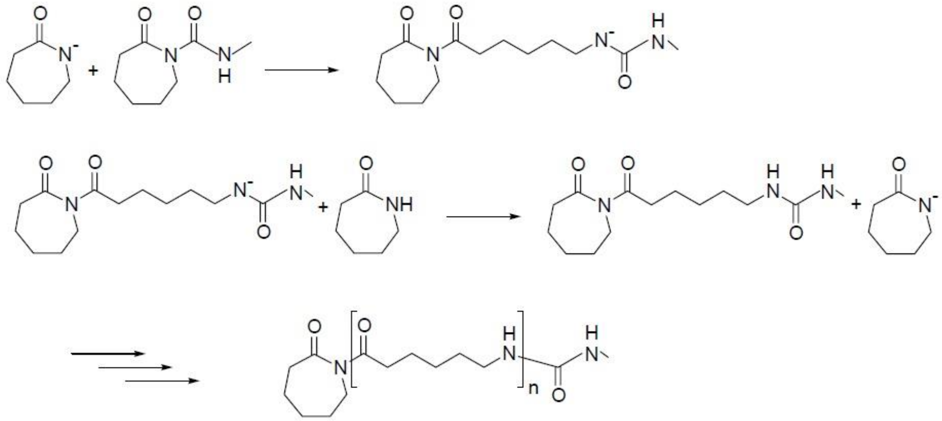

Figure S1. Mechanism of the synthesis of polyamide 6 by anionic ring-opening polymerization [3].

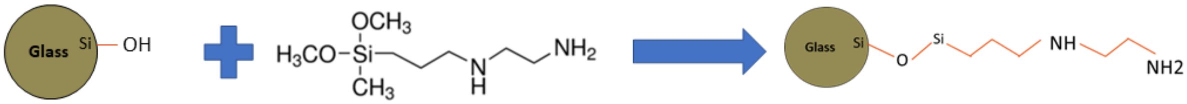

Figure S2. Grafting of 3-(2-aminoethylamino)propyltrimethoxysilane (AEAPTMS) on the glass particles surface.

2. Effect of the glass beads size distribution on the specific surface measurement

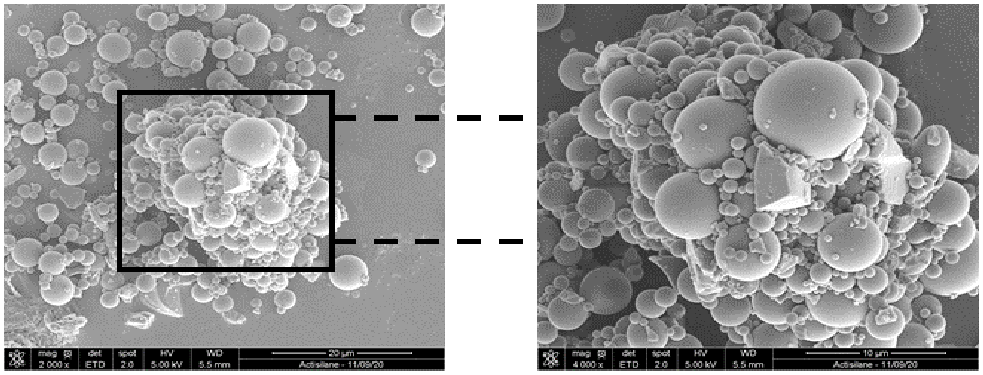

Figure S3. SEM micrographs of glass spheres used in the present study showing the aggregates forming the microporosity.

SEM micrographs (**Figure S3**) show that the glass powder used in the present study consists of particles with an average diameter of 4 µm forming aggregates, which generates very fine microporosities not detectable by BET analysis. Consequently, the specific surface measured by

this technique is lower than the real specific surface, which considerably increases the apparent hydroxyl density value.

3. Fourier transform infrared spectroscopy measurements

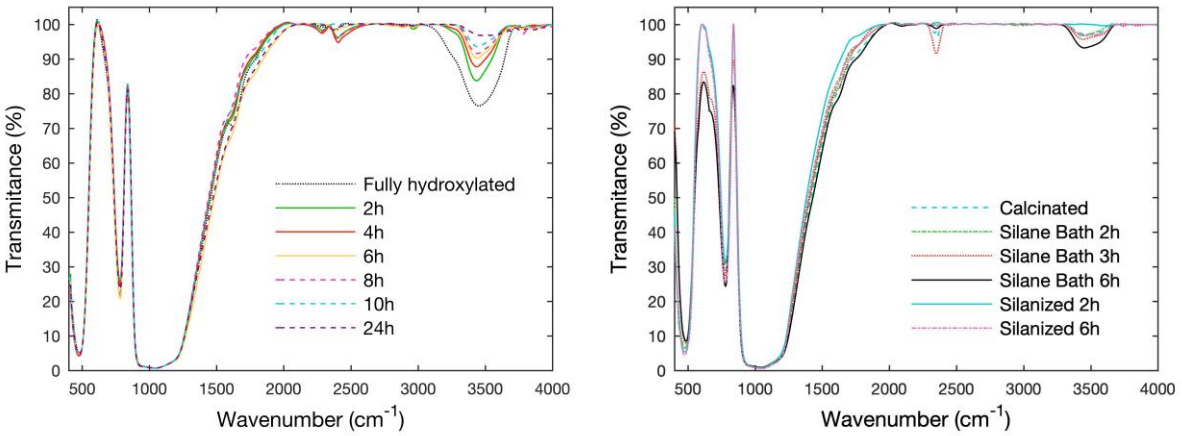

Figure S4. Evolution of the infrared transmission spectra of (a) initially rehydroxylated glass particles, as a function of the subsequent calcination time at 450◦C, (b) glass particles calcinated for 10 h and subsequently partially rehydroxylated in the silanization solution without the silane (silane bath) and with the silane (silanized) for different durations.

4. Comparison of the rehydroxylation process between a 10% HCl solution, and a pH 4-5 acetic acid solution.

After calcination, controlling the rehydroxylation process during the silanization step is crucial in order to avoid an excessive regeneration of –OH at the particles' surface, which can slow down or inhibit the polymerization/crystallization processes. The methodology that is often used in the literature for the rehydroxylation of glass particles is by immersion in a hydrochloric acid (HCl) solution.1,2. However, this technique may regenerate an excessive number of surface -OH. Therefore, another approach consists in simultaneously regenerating (moderately) the hydroxyls at the particles surface, and grafting the silane agent at the surface. This protocol aims at generating a sufficient number of OH for the silane grafting process, without regenerating residual OH. Therefore, comparing the rehydroxylation process in HCl 10 %, and in the silane treatment bath (aqueous solution adjusted at pH 4-5 with acetic acid, but without the silane agent at this step) is pertinent in order to select the most appropriate approach for the silane grafting step, without negatively influencing the polymerization/crystallization processes. The glass particles were thus calcinated for 24 h at 450 ◦C to eliminate a maximum number of hydroxyl groups. Half of the calcinated particles were then immersed in the 10% HCl solution, and the second half was added to the aqueous solution adjusted at pH 4-5 with acetic acid (silane treatment bath, without the silane), for 3 h in both cases.

The TGA results (Table S1) show that the mass concentration of regenerated hydroxyls after 3 h is significantly higher in 10 % HCl. In contrast, the silanization treatment bath yields moderate rehydroxylation after 3 h, and the mass concentration of regenerated hydroxyls remains low and

sufficient for adequate silane grafting. These results were confirmed by FTIR spectroscopy (Figure S2). The spectra show that the particles rehydroxylated for 3 h in 10% HCl present a peak with a large band similar to the case of the fully rehydroxylated particles - after 3 h, the maximum -OH concentration at the surface has already been reached. However, the particles rehydroxylated in the silanization bath show a peak with features similar to the particles calcinated for 24 h, with a slightly higher intensity due to the regeneration of the hydroxyls.

Table S1. TGA weight loss and hydroxyls mass concentration for glass particles calcinated for 24 h, and calcinated particles subsequently rehydroxylated in the silanization solution (aqueous solution adjusted at pH 4-5 with acetic acid, without the silane), or in 10 % HCl, for 3 h.

Sample code	TGA weight loss (± 0.01 %)	Hydroxyls mass concentration (mmol $OH.g^{-1}$)
Calcinated 24 h	0	0
Silanization solution 3 h	0.17	0.19 ± 0.01
10% HCl solution 3 h	0.69	0.77 ± 0.01

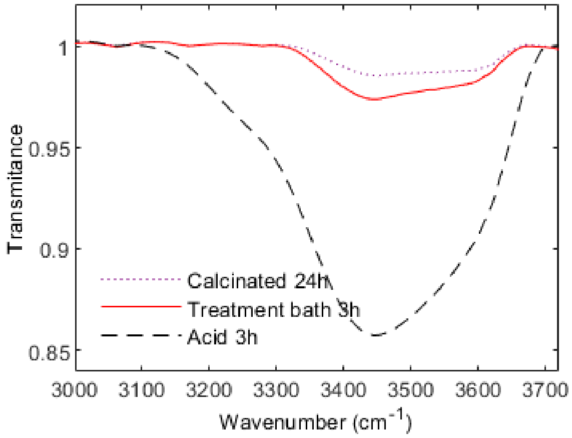

Figure S5. Comparison of FTIR spectra for the glass particles calcinated for 24 h, and particles rehydroxylated respectively in the silanization solution (aqueous solution adjusted at pH 4-5 with acetic acid, without the silane), and in 10 % HCl, for 3 h.

The hydroxyls concentration measured by TGA, and the -OH peak intensity obtained by FTIR, for the particles rehydroxylated in 10 % HCl, are very high compared to the particles rehydroxylated in the silanization solution, for the same treatment time. This shows that the regeneration rate of hydroxyls in 10 % HCl is very high and might not be suitable for the synthesis of anionic PA6. Therefore, the use of an aqueous solution at pH 4-5 for the silanization process appears to be more appropriate.

5. Differential Scanning Calorimetry measurements

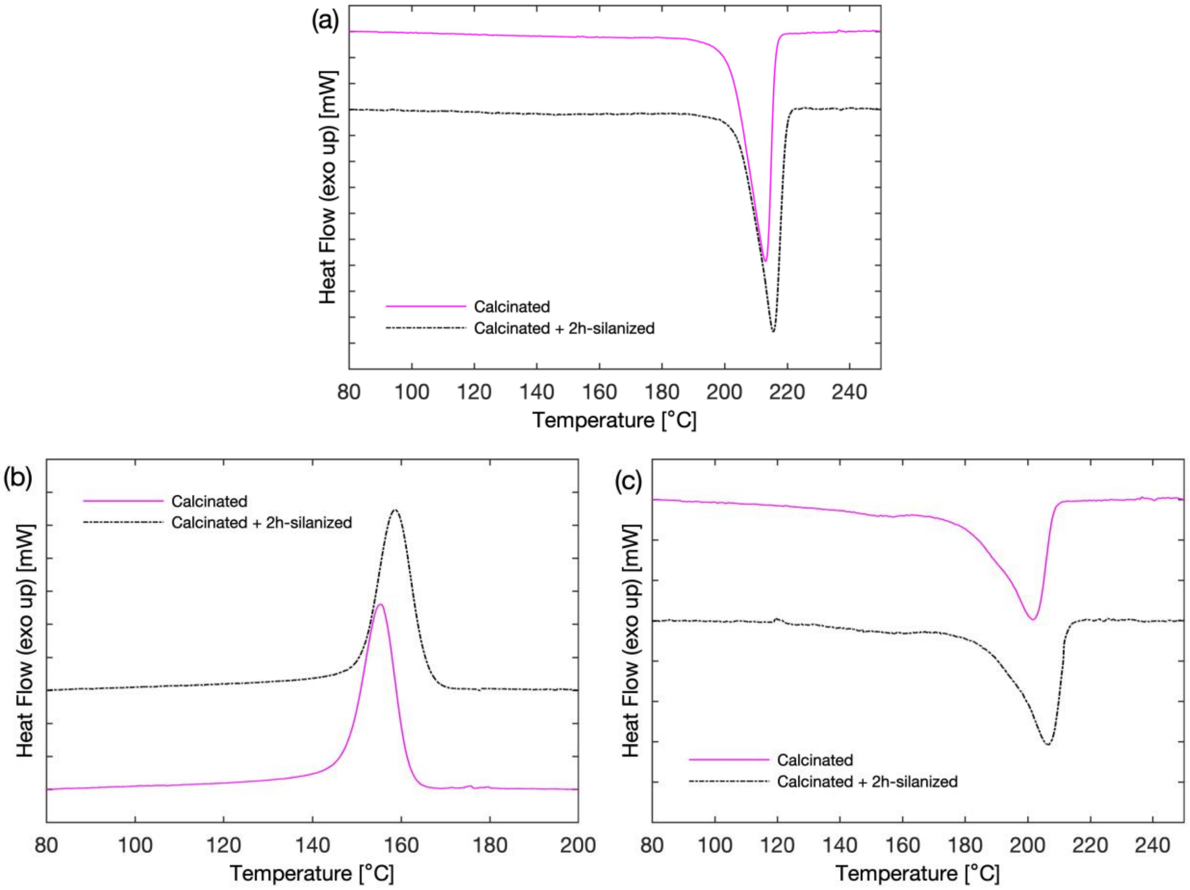

Figure S6. DSC curves of heating and cooling steps subsequent to isothermal syntheses at 180 ◦C, of composites synthesized with strictly calcinated particles, and particles silanized for 2 h. (a) Melting peak at 10°C/min after synthesis; (b) Crystallization peak from melt at -10°C/min; (c) Melting peak at 10°C/min of melt crystallized composites.

References

1. K. van Rijswijk, J. J. E. Teuwen, H. E. N. Bersee, et A. Beukers, « Textile fiber-reinforced anionic polyamide-6 composites. Part I: The vacuum infusion process », Composites Part A: Applied Science and Manufacturing, vol. 40, no 1, p. 1-10, janv. 2009

2. Gonzalez-Benito, J., Cabanelas, J.C., Aznar, A.J., Vigil, M.R., Bravo, J. and Baselga, J. (1996), Surface characterization of silanized glass fibers by labeling with environmentally sensitive fluorophores. J. Appl. Polym. Sci., 62: 375-384. https://doi.org/10.1002/(SICI)1097-4628(19961010)62:2<375::AID-APP12>3.0.CO;2-0.

3. Sever, K., Seki, Y., Tavman, I.H., Erkan, G. and Cecen, V. (2009), The structure of γ-glycidoxypropyltrimethoxysilane on glass fiber surfaces: Characterization by FTIR, SEM, and contact angle measurements. Polym Compos, 30: 550-558. https://doi.org/10.1002/pc.20585.
